# Different approach, similar outcomes: the impact of surgical access routes in minimally invasive cardiac surgery on enhanced recovery after surgery

**DOI:** 10.3389/fcvm.2024.1412829

**Published:** 2024-07-01

**Authors:** Sarah Berger Veith, Theresa Holst, Sahab Erfani, Julia Pochert, Christian Dumps, Evaldas Girdauskas, Sina Stock

**Affiliations:** ^1^ Department of Cardiothoracic Surgery, Augsburg University Hospital, Augsburg, Germany; ^2^ Department of Anesthesiology and Surgical Intensive Care Medicine, Augsburg University Hospital, Augsburg, Germany

**Keywords:** enhanced recovery after surgery (ERAS), enhanced recovery after cardiac surgery (ERACS), minimally invasive cardiac surgery (MICS), anterolateral mini-thoracotomy, partial sternotomy, mini-sternotomy, outcome

## Abstract

**Objectives:**

Enhanced recovery after surgery (ERAS) is a growing phenomenon in all surgical disciplines and aims to achieve a faster functional recovery after major operations. Minimally invasive cardiac surgery (MICS) therefore integrates well into core ERAS values. Surgical access routes in MICS include right anterolateral mini-thoracotomy (MT) as well as partial upper mini-sternotomy (PS). We seek to compare outcomes in these two cohorts, both of which were enrolled in an ERAS scheme.

**Methods:**

358 consecutive patients underwent MICS and perioperative ERAS at our institution between 01/2021 and 03/2023. Patients age >80 years, with BMI > 35 kg/m², LVEF ≤ 35%, endocarditis or stroke with residuum were excluded. Retrospective cohort analysis and statistical testing was performed on the remaining 291 patients. The primary endpoint was successful ERAS, secondary endpoints were the occurrence of major bleeding, ERAS-associated complications (reintubation, return to ICU) as well as access-related complications (wound infection, pleural and pericardial effusions).

**Results:**

170 (59%) patients received MT for mitral and/or tricuspid valve surgery (*n* = 162), closure of atrial septal defect (*n* = 4) or resection of left atrial tumor (*n* = 4). The remaining 121 (41%) patients had PS for aortic valve repair/replacement (*n* = 83) or aortic root/ascending surgery (*n* = 22) or both (*n* = 16). MT patients’ median age was 63 years (IQR 56–71) and 65% were male, PS patients’ median age was 63 years (IQR 51–69) and 74% were male. 251 (MT 88%, PS 83%, *p* = 0.73) patients passed through the ERAS program successfully. There were three instances of reintubation (2 MT, 1 PS), and three instances of readmission to ICU (2 MT, 1 PS). Bleeding requiring reexploration occurred six times (3 MT, 3 PS). There was one death (PS), one stroke (MT), and one myocardial infarction requiring revascularization (MT). There were no significant differences in any of the post-operative outcomes recorded, except for the incidence of pericardial effusions (MT 0%, PS 3%, *p* = 0.03).

**Conclusions:**

Despite different surgical access routes and underlying pathologies, results in both the MT and the PS cohort were generally comparable for the recorded outcomes. ERAS remains safe and feasible in these patient groups.

## 1 Introduction

Enhanced recovery after surgery (ERAS) is a growing phenomenon in all surgical disciplines, including cardiac surgery (1). It is an interdisciplinary and multimodal protocol which involves all members of the healthcare team and centers the patient. The goal is to establish interventions in the pre-, intra-, and postoperative phases that contribute to a faster and more complete functional recovery after major surgery. Recommendations often focus on opioid-sparing analgesia, judicious intravenous fluid use and transfusion, improved perioperative nutrition and intensive physiotherapy (1–3). In cardiac surgery, adoption of ERAS is complicated by the highly invasive nature of the procedures and the substantial risk for postoperative morbidity. Nonetheless, numerous centers have established ERAS programs in cardiac surgery (4–8), following the recommendations of the ERAS cardiac society (ERACS) which was founded in 2017 (9). With the introduction of minimally invasive cardiac surgery (MICS) in the late 1990s and early 2000s (10–12), methods were pioneered that likewise aimed to reduce surgical trauma and therefore improve postoperative recovery. Surgical access routes in MICS include right anterolateral mini-thoracotomy (MT) and partial upper mini-sternotomy (PS). In 2022, in a review of all isolated valve procedures in the German Society for Thoracic and Cardiovascular Surgery registry, 40% of aortic valve surgeries took place via PS and 59% of mitral valve surgeries took place via MT (13).

Beginning in 2021, our center began to develop a general ERAS program aimed at the broad majority of patients receiving MICS based on the ERACS recommendations. Our center’s participation in the INCREASE trial (14) provided some important building blocks to this project. The institutional ERAS program is also partly influenced by the principles published by Kubitz et al. (15), which place a high value on psychosocial and physiotherapeutic interventions. Additionally, the use of minimally invasive access routes enabled uncompromised chest stability, leading to improved respiratory mechanics immediately after surgery. This, in turn, made routine on-table extubation possible for all patients who had received MICS and was established as a cornerstone of our ERAS program. Only very minor alterations were made to the ERAS protocol to account for the underlying surgery or access route, primarily concerning the use of different forms of regional anesthesia. Otherwise, all minimally invasive valve patients were treated according to the same perioperative algorithm.

However, the underlying diseases treated via MT and PS differ—and so do the effects of the specific type of surgical trauma between the access routes: In comparisons of patients receiving aortic valve replacement via either PS or MT, there is some evidence suggesting that MT results in shorter ventilation times, faster mobilization and earlier discharge from hospital (16, 17), thereby making it possible that MT offers better anatomical prerequisites for ERAS than PS. Our current ERAS program does not take into consideration either the type of access used, nor the pathology treated. Patients receiving MICS are all treated according to the same considerations. If, in fact, MT or PS patients had a propensity towards worse recovery or towards access- or pathology-specific complications, adapting the ERAS program to either the type of access or the treated disease would become necessary.

Therefore, the aim of this study is to investigate whether patients receiving MT group and those receiving PS group as part of a unified ERAS program achieve the same degree of recovery and whether any complications during that recovery are more attributable to the underlying pathologies or to the type of surgical access.

## 2 Methods

### 2.1 Study design and patient selection

We conducted a retrospective observational study of adult patients receiving MICS and deemed suitable for our institution's ERAS program. The study was conducted in accordance with the Declaration of Helsinki (2008) and ethics approval was received from the institutional review board at our institution (Institutional Review Board Ludwig Maximilian University Munich, Germany, project number 23-0915). All patients who received MICS between 01/2021 and 03/2023 and who had been marked preoperatively for our ERAS scheme were included in a retrospective database. Exclusion criteria for our ERAS program were emergency procedures, redo operations, those patients unwilling to participate in ERAS and those patients unable to achieve compliance with ERAS interventions due to neurological or physical limitations (e.g., known alcohol use disorder, cognitive impairment, inability to walk preoperatively). Due to the retrospective nature of this study, no written consent was required. The selected patients underwent standardized data collection for demographic, intra- and postoperative data. For the purposes of this analysis, we excluded patients above the age of 80 years, those with a body mass index >35 kg/m^2^, left ventricular ejection fraction ≤35%, endocarditis or stroke with residuum. This was undertaken to better isolate the impact of the surgical access route on outcomes and to remove outliers. Retrospective cohort analysis was performed on the remaining patients.

The primary endpoint was successful ERAS, which was defined as having reached at least three of five ERAS goals: (1) on-table extubation, (2) transfer to intermediate instead of intensive care unit (ICU), (3) early physiotherapy starting on the day of surgery, (4) transfer to the ward within 24 h postoperatively and (5) discharge home or to a rehab center within 7 days. Secondary endpoints were possibly ERAS-associated complications reintubation and readmission to ICU, as well as bleeding requiring reexploration, pericardial/pleural effusions and wound healing complications.

### 2.2 Surgical technique

Mitral valve and/or tricuspid valve surgery, atrial septal defect closure and myxoma resection was addressed through right anterolateral mini-thoracotomy, which was achieved by a right anterolateral incision in the fourth intercostal space. The surgery proceeds without rib-spreading and through use of fully endoscopic technique.

Aortic valve surgery, ascending aortic as well as aortic root procedures and combinations thereof were performed using a partial upper J mini-sternotomy in the third or fourth intercostal space.

Both approaches aim to maintain the stability of the chest and, therefore, to enable on-table extubation and early ambulation.

### 2.3 The institutional ERAS program

Our institution's ERAS program is designed for principally all comers receiving minimally invasive valve or aortic surgery. Unless patients were individually deemed very high surgical risk, patients receiving MICS for valve or aortic surgery were treated with ERAS principles. Selection of patients was accomplished on a case-by-case basis by a senior surgeon.

The core tenets of the ERAS program include:

(I) Prehabilitation including nutritional, psychological and physical optimization, as well as structured interviewing before admission for surgery. This is facilitated by an ERAS nurse, an advanced practice nurse who coordinates preoperative clinic visits for patients. There, patients are seen by a psychologist, a physical therapist, a cardiac surgeon, a cardiac anesthesiologist and the ERAS nurse for thorough assessment and preoperative preparation.
(II) Use of minimally invasive surgical approach (i.e., MT or PS).
(III) On-table extubation and fast-track recovery through the intermediate care unit (IMC). Goals are the transfer from IMC to the general ward within 24 h postoperatively as well as discharge home or to a cardiac rehabilitation facility within five to seven days.
(IV) Intensive physiotherapy and mobilization. Concrete goals are standing up from bed on the day of surgery and ambulating on postoperative day (POD) 1. Daily physiotherapy is provided in addition to mobilization from bed by the nursing staff, with patients ideally being able to climb stairs independently by the time of discharge.

These goals are supported by standardized postoperative nausea and vomiting (PONV) prophylaxis, opioid-sparing multimodal analgesia (e.g., including routine regional anesthetic procedures such as serratus anterior plane block for patients receiving MT and parasternal block for patients receiving PS) and motivational interviewing, which aims to help the patient activate their psychological coping skills and empower them to seek an active role in their care. Patients are further encouraged to wear their own clothing, preferably regular daytime dress, as soon as they are transferred to the ward to decrease the psychological alienation major surgery and hospitalization can engender.

### 2.4 Statistical analysis

Results were tested for normality using the Shapiro–Wilk test. Data are presented as median and interquartile range or absolute and relative frequencies. Mann–Whitney *U* test and Fisher's Exact test were used for non-parametric unpaired data. Results from statistical tests were regarded as significant when *p* < 0.05. Analysis was performed through GraphPad Prism Version 10.2.1 (GraphPad Software, Boston, MA, USA).

## 3 Results

### 3.1 Demographics

Between January 2021 and March 2023, 358 patients were deemed suitable for ERAS in the setting of MICS at our institution and agreed to participate. The institution's ERAS program was established in January 2021, there being 175 cases in 2021, 145 cases in 2022 and 38 cases in the first quarter of 2023. After applying the exclusion criteria mentioned above, 291 patients remained. Patients were separated into two groups according to the planned minimally invasive surgical access route—either MT or PS. The demographic and operative data of both groups are demonstrated in Tables 1,2.

**Table 1:** Demographic data for both the mini-thoracotomy (MT) and mini-sternotomy (PS) groups.

*n* = 291	Mini-thoracotomy *n* = 170	Mini-sternotomy *n* = 121	*p*-value
Age (years)	63 (56–71)	63 (51–69)	0.20
Sex, male	110 (65%)	89 (74%)	0.13
BMI (kg/m^2^)	25 (23–27)	26 (24–29)	**<0**.**001**
Atrial fibrillation/flutter	58 (34%)	13 (11%)	**<0**.**001**
Coronary artery disease	32 (19%)	24 (20%)	0.88
LVEF (%)	60 (58–61)	60 (55–60)	0.07
Diabetes mellitus	13 (8%)	11 (9%)	0.67
Lung disease	21 (12%)	9 (7%)	0.24
Severe renal impairment^a^	20 (12%)	9 (7%)	0.24
Stroke	15 (9%)	6 (5%)	0.26
EuroSCORE II (%)	1.0 (0.7–1.5)	1.0 (0.7–1.5)	0.61
STS PROM score (%)	(0.4–1.1)	0.8 (0.5–1.1)	0.08

Data presented as median (IQR) or absolute and relative frequencies. *p*-values derived from Mann–Whitney *U* test or Fisher's exact test. *p*-values considered significant if <0.05.

BMI, body mass index; LVEF, left ventricular ejection fraction; STS, society of thoracic surgeons; PROM, predicted risk of mortality.

^a^ Creatinine Clearance <55 ml/min, no dialysis patients in this cohort.

Bold values denote statistically significant *p*-values.

**Table 2:** Operative data for the mini-thoracotomy (MT) and mini-sternotomy (PS) groups.

Access	Operation	
Mini-thoracotomy (*n* = 170)	*Mitral valve repair/replacement*	153 (90%)
	*ASD repair*	4 (2%)
	*Myxoma resection*	4 (2%)
	*Tricuspid valve repair/replacement*	2 (1%)
	*Combined MV/TV surgery*	5 (3%)
	*Combined MV and ASD repair*	2 (1%)
Mini-sternotomy (*n* = 121)	*Aortic valve replacement*	68 (56%)
	*Aortic valve repair*	15 (12%)
	*David/Bentall procedure*	17 (14%)
	*Ascending aortic aneurysm repair*	6 (5%)
	*Combined aortic valve and AAA repair*	15 (12%)

AAA, ascending aortic aneurysm; ASD, atrial septal defect; MV, mitral valve; TV, tricuspid valve.

MT patients’ median age was 63 years (IQR 56–71) and 65% were male, PS patients’ median age was 63 years (IQR 51–69) and 74% were male. Statistically significant differences at baseline between the groups were observed for the prevalence of atrial fibrillation as well as for body mass index.

In 170 (59%) patients MT was performed for mitral and/or tricuspid valve surgery (*n* = 162), closure of atrial septal defect (*n* = 4) or resection of left atrial tumor (*n* = 4). The remaining 121 (41%) patients had PS for aortic valve repair/replacement (*n* = 83) or aortic root/ascending surgery (*n* = 23) or both (*n* = 15), see Table 2. Of the patients with preexsisting atrial fibrillation, 66% of patients receiving MT received left atrial appendage (LAA) closure and endoatrial cryoablation and a further 19% received only LAA closure. Those with preexisting atrial fibrillation in the PS group received LAA closure in 46% of cases.

### 3.2 Major postoperative complications

There were three total major adverse cardiovascular events (MACE) in both groups: one mortality (PS; left main coronary artery occlusion after David procedure), one stroke (MT; posterior inferior cerebellar artery stroke) and one myocardial infarction requiring revascularization (MT; circumflex artery occlusion after mitral valve repair). Instances of bleeding requiring reexploration occurred a total of three times in each group (MT 2%, PS 3%, *p* = 0.70): One acute and two subacute hemothoraces in the MT group, three episodes of cardiac tamponade on POD 1 in the PS group.

### 3.3 ERAS achievement

ERAS success as well as potentially access-related complications are listed in Table 3. In 251 (MT 88%, PS 83%) patients the ERAS program was successful, meaning they achieved three or more ERAS items (see 2.1). There were no significant differences in the degree of ERAS achievement between the groups (*p* = 0.73). Potentially ERAS-related complications were rare: There were three instances of reintubation (1 MT, 2 PS, *p* = 0.57), and four instances of readmission to ICU (1 MT, 3 PS, *p* = 0.31) for hypotension or respiratory insufficiency. Postoperative length of stay (LOS) was a median of 6 days in both groups (both: IQR 5–8; *p* = 0.82).

**Table 3:** Postoperative outcomes between the mini-thoracotomy (MT) and the mini-sternotomy (PS) groups.

*n* = 291	Mini-thoracotomy *n* = 170	Mini-sternotomy *n* = 121	*p*-value
ERAS success (≥3 points)	150 (88%)	100 (83%)	0.73
0	9 (5%)	9 (7%)	
1	5 (3%)	2 (1%)	
2	6 (4%)	9 (7%)	
3	26 (15%)	16 (13%)	
4	42 (25%)	26 (21%)	
5	82 (48%)	58 (48%)	
Reexploration for bleeding	3 (2%)	3 (3%)	0.70
Pleural effusion^a^	19 (11%)	17 (14%)	0.48
Pericardial effusion^b^	0 (0%)	4 (3%)	**0**.**03**
Primary wound infections^c^	0 (0%)	0 (0%)	-
Post-operative atrial fibrillation	47 (28%)	29 (24%)	0.50
Return within 3 months for			
Inguinal seroma^c^	4 (2%)	1 (1%)	0.41
Wound infection^c^	4 (2%)	4 (3%)	0.72

Data presented absolute and relative frequencies. *p*-values derived from Fisher's exact test. *p*-values considered significant if < 0.05. ERAS success was scored in five items (see 2.1), with successful ERAS being defined as having reached ≥3 goals.

ERAS: enhanced recovery after surgery.

^a^ Requiring intervention, either diuretic therapy or drainage.

^b^ Requiring intervention, either surgical or percutaneous evacuation.

^c^ Requiring vacuum-assisted closure therapy or revision.

Bold values denote statistically significant *p*-values.

### 3.4 Surgical access-related complications

**Figure 1:**
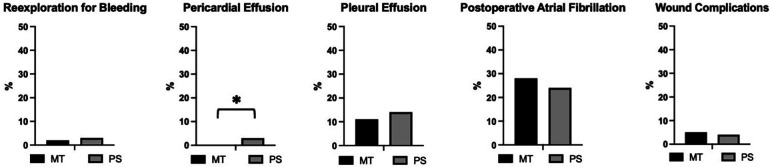
Relative frequency of access-related complications after mini-thoracotomy (MT) or mini-sternotomy (PS) in percent. Reexploration for bleeding (3/170 of MT and 3/121 of PS patients). Pericardial effusion (0/170 of MT and 4/121 of PS patients). Pleural effusion (19/170 of MT and 17/121 of PS patients). Postoperative atrial fibrillation (47/170 of MT and 29/121 of PS patients). Wound complications requiring surgical intervention (8/170 of MT and 5/121 of PS patients). Statistically significant difference between the groups present only for pericardial effusions (*p* = 0.03, indicated by *). *P*-values derived from Fisher's Exact test and considered significant when <0.05.

Access-related complications are illustrated in Table 3; Figure 1. Bleeding, as mentioned above, was rare. There was an 11% (MT) and 14% (PS) incidence of postoperative pleural effusions needing either significant diuretic therapy or drainage. Post-operative atrial fibrillation was fairly common in both groups (MT 28%, PS 24%), without any significant difference. There were four late pericardial effusions (>5 days postoperatively) only in the PS group. All four patients had to be readmitted for percutaneous or surgical decompression of the pericardium. This was statistically significantly different from the MT group, in which no pericardial effusions requiring intervention occurred at all. There was no primary wound infection or dehiscence requiring intervention during the initial hospitalization. A total of eight patients in the MT group (5%) and five patients in the PS group (4%) had to be rehospitalized for wound infections requiring intervention, either surgical revision or VAC therapy. There was no statistical difference between the groups for this metric.

## 4 Discussion

Achievement of ERAS in this cohort was independent of the surgical access route used, with very good safety outcomes in both the MT and PS groups. There was a small but significant difference in the incidence of late pericardial effusions requiring drainage, favoring the MT group. Otherwise, no statistically significant differences were observed for any of the recorded outcomes, including reexploration for bleeding, post-operative atrial fibrillation, pleural effusions and wound complications. One of the points of interest in this analysis is the comparison of two principally different cohorts of patients—those with aortic valve and aortic pathology in the PS group and those with atrioventricular valve or atrial pathology in the MT group—and whether one unified ERAS program was sufficient to account for both the different forms of surgical trauma and the underlying disease processes. In several prior studies, mixed populations of patients receiving both MT and PS for various pathologies were treated within the same perioperative care regime without any further analysis if this was equally appropriate for both these groups of patients (6, 15, 18). We sought to address the question whether these patients can indeed be treated within the same ERAS protocol or whether, due to either fundamental differences in the patient populations or the type of procedure performed, an ERAS protocol would have to contain specific considerations for these elements in order to be effective.

### 4.1 Postoperative recovery and length of stay

Two recent meta-analyses showed that mitral valve surgery was associated with shorter ICU stays and shorter hospitalization overall when performed via MT rather than full sternotomy (19, 20). Sündermann et al. report, in their meta-analysis of 27 trials (4,948 patients), an average in-hospital length of stay after mitral valve surgery of 8 ± 3 days after MT and 9 ± 3 days after full sternotomy (20). Likewise, a large recent meta-analysis comparing different access routes for aortic valve replacement (AVR), including MT, PS and full sternotomy, also showed significantly shorter ICU stays and shorter hospitalization associated with PS compared to full sternotomy, but no difference between MT and PS (21). This suggests that, on aggregate, recovery after MICS for both mitral and aortic valve surgery is faster than after conventional full sternotomy, which is in accordance with the importance we have placed on a minimally invasive surgical access route in our ERAS program. Direct comparisons of recovery after surgery via MT vs. PS exist primarily for AVR, since this procedure can be accomplished via both routes: A 2020 systematic review patients receiving AVR either via PS or MT found, in an analysis of five trials, no difference in ICU length of stay, but shorter hospitalization in the MT group (22), possibly suggesting faster postoperative recovery. This was not reflected in our data, where recovery, as indicated both by the ERAS score and LOS, was not significantly different between the groups. Our data therefore addresses the knowledge gap in the question of if there is additional recovery benefit to MT when compared, not to full sternotomy, but to PS.

### 4.2 Bleeding

There is heterogenous data regarding reexploration for bleeding and its relationship to the type of surgical access: The above mentioned systematic review by El-Andari et al. found a significantly higher rate of bleeding requiring reexploration in patients receiving AVR via MT compared to PS (22). Conversely, in one retrospective, propensity-matched analysis of AVR via PS or MT published after the systematic review, Bakhtiary et al. (16) found a significantly higher rate of reoperation for bleeding in their PS group than in their MT group. This is interesting, especially since bleeding in MT in our cohort was exclusively from the chest wall, and therefore access-related, and not from cardiac structures themselves. It seems possible, though, that primary cardiac bleeding might be more common in total after AVR due to aortotomy than after mitral valve surgery. In our data, there was no difference between the groups for the rate of reexploration due to bleeding. It is worth noting, however, that 2/3 bleeding events and 1/4 of the pericardial effusions in the PS group were in patients having received aortic root repairs. Aortic root procedures were, due to their high surgical risk, deemed likely unsuitable for on-table extubation in the ERACS society consensus statement (3). In this very small cohort of patients (*n* = 17), we have not found aortic root procedures to be prohibitive for on-table extubation, but the increased postoperative bleeding risk is noteworthy. The instances of reintubation mentioned above in the PS group occurred in patients after AVR.

### 4.3 Retained blood syndrome and post-pericardiotomy syndrome

An aspect to consider further would be placement of chest drains in PS: Often, only one pericardial drain was placed anteriorly, leaving the dorsocaudal aspect of the pericardium possibly without sufficient drainage and therefore possibly contributing to retained blood syndrome (23). This could offer an explanation as to the higher (if still very low) incidence of subacute pericardial effusions requiring intervention in the PS group. These were all “late” effusions, i.e., >5 days postoperatively, presenting as slowly progressive. They were all either partly or entirely serous upon removal, and therefore not primarily due to bleeding. It is also possible that this difference was driven instead by a higher incidence of post-pericardiotomy syndrome (24), which is thought to be more common in aortic and aortic valve surgery. There is an intersection between post-pericardiotomy syndrome and retained blood, however, since retained blood is believed to be an important trigger for the inflammatory, exudative reaction of post-pericardiotomy syndrome (25). Further research is needed in this area. Other features of retained blood syndrome, such as postoperative atrial fibrillation and pleural effusions were equal in both groups.

### 4.4 Wound healing

The concern for more wound healing complications after PS than after MT, which is often considered to be cosmetically preferrable, has so far not borne out in the prior literature (16, 21). Our data reflects this: There were no instances of primary wound dehiscence or infection in the initial hospital stay. 5% of patients in the MT group and 4% in the PS group had to be rehospitalized for inguinal seromas or wound infections requiring intervention, with no statistically significant differences observed.

### 4.5 Limitations

A limitation of this analysis is its single-center, retrospective design and the unmatched data between the groups, although the baseline demographic characteristics are comparable. Statistically significant differences were present for atrial fibrillation—likely due to the corresponding incidence of mitral valve disease—and body mass index. The absolute difference in body mass index between the groups was, while statistically relevant, clinically insignificant with a median body mass index of 25 kg/m^2^ in the MT group and 26 kg/m^2^ in the PS group. Furthermore, patient selection for the ERAS program is rigorous, excluding patients with certain individual risk factors that were deemed incompatible with a successful application of ERAS [see (2.3)]. In a second step, certain higher risk features such as active endocarditis and reduced left ventricular function were excluded to not confound bleeding- and recovery-associated outcomes between the groups. Therefore, our data may not be applicable to patient cohorts with higher risk features.

## 5 Conclusion

In this study, the success of fast-track ERAS was similar regardless of the surgical access route, demonstrating good safety outcomes in both groups. Although there was a slight disparity in the incidence of late pericardial effusions favoring the MT group, no other statistically significant differences were observed, including rates of reexploration for bleeding, post-operative atrial fibrillation, pleural effusions, and wound complications. This analysis also highlights that a non-specific fast-track ERAS program can be effective in managing patients undergoing different cardiac surgical procedures and addressing various underlying disease processes.

## Data Availability

The raw data supporting the conclusions of this article will be made available by the authors, without undue reservation.
